# Clinical utility of target amplicon sequencing test for rapid diagnosis of drug-resistant *Mycobacterium tuberculosis* from respiratory specimens

**DOI:** 10.3389/fmicb.2022.974428

**Published:** 2022-09-09

**Authors:** Kenneth Siu-Sing Leung, Kingsley King-Gee Tam, Timothy Ting-Leung Ng, Hiu-Yin Lao, Raymond Chiu-Man Shek, Oliver Chiu Kit Ma, Shi-Hui Yu, Jing-Xian Chen, Qi Han, Gilman Kit-Hang Siu, Wing-Cheong Yam

**Affiliations:** ^1^Department of Microbiology, Queen Mary Hospital, The University of Hong Kong, Pokfulam, Hong Kong SAR, China; ^2^Department of Health Technology and Informatics, The Hong Kong Polytechnic University, Kowloon, Hong Kong SAR, China; ^3^KingMed Diagnostics, Science Park, Kwun Tong, Hong Kong SAR, China; ^4^Guangdong-Hong Kong-Macao Joint Laboratory of Respiratory Infectious Disease, Guangzhou, China; ^5^Guangzhou KingMed Diagnostics Group, Guangzhou, China

**Keywords:** *Mycobacterium tuberculosis*, next-generation sequencing, drug resistance, diagnosis, MDR-TB

## Abstract

An in-house-developed target amplicon sequencing by next-generation sequencing technology (TB-NGS) enables simultaneous detection of resistance-related mutations in *Mycobacterium tuberculosis* (MTB) against 8 anti-tuberculosis drug classes. In this multi-center study, we investigated the clinical utility of incorporating TB-NGS for rapid drug-resistant MTB detection in high endemic regions in southeast China. From January 2018 to November 2019, 4,047 respiratory specimens were available from patients suffering lower respiratory tract infections in Hong Kong and Guangzhou, among which 501 were TB-positive as detected by in-house IS6110-qPCR assay with diagnostic sensitivity and specificity of 97.9 and 99.2%, respectively. Preliminary resistance screening by GenoType MTBDR*plus* and MTBDR*sl* identified 25 drug-resistant specimens including 10 multidrug-resistant TB. TB-NGS was performed using MiSeq on all drug-resistant specimens alongside 67 pan-susceptible specimens, and demonstrated 100% concordance to phenotypic drug susceptibility test. All phenotypically resistant specimens with dominating resistance-related mutations exhibited a mutation frequency of over 60%. Three quasispecies were identified with mutation frequency of less than 35% among phenotypically susceptible specimens. They were well distinguished from phenotypically resistant cases and thus would not complicate TB-NGS results interpretations. This is the first large-scale study that explored the use of laboratory-developed NGS platforms for rapid TB diagnosis. By incorporating TB-NGS with our proposed diagnostic algorithm, the workflow would provide a user-friendly, cost-effective routine diagnostic solution for complicated TB cases with an average turnaround time of 6 working days. This is critical for timely management of drug resistant TB patients and expediting public health control on the emergence of drug-resistant TB.

## Introduction

Tuberculosis (TB) caused by *Mycobacterium tuberculosis* (MTB) accounts for an estimated 10 million new cases and 1.5 million TB-related deaths annually (World Health Organization, 2021). This situation was further complicated with drug-resistant TB emergence. Globally, around 3.4% of new TB cases and 18% of previously treated cases were classified as multidrug-resistant TB (MDR-TB) with resistance to both rifampicin (RIF) and isoniazid (INH), or RIF mono-resistant (RR-TB) strains. Approximately 6.2% of global MDR-TB/RR-TB cases could be further classified as extensively drug-resistant (XDR-TB) strains with additional resistance to fluoroquinolones and at least one additional Group A anti-TB drug (bedaquiline or linezolid; World Health Organization, 2021). According to a national survey back in 2007, approximately 10% of TB cases in China were classified as MDR-TB (Zhao et al., 2012). To ensure a better control of the global pandemic, a rapid, accurate and cost-effective diagnostic platform for TB detection and subsequent drug resistance identification is required.

Previous studies have explored the use of next-generation sequencing (NGS) for TB drug resistance determination, and the platform has proved its capability as a diagnostic tool for TB outbreak control and drug susceptibility predictions in clinical isolates (Walker et al., 2017; Satta et al., 2018; Comin et al., 2020). While these studies have demonstrated the feasibility of NGS in TB drug resistance diagnosis, the sequencing platforms are either obsoleted (Colman et al., 2016), or the study only focused on AFB positive specimens with limited variability in drug resistance patterns (Smith et al., 2020). More importantly, as most platform focused on whole genome sequencing data, high-quality genomic DNA prepared from MTB isolates are usually required (Lam et al., 2021; Olawoye et al., 2021). The long turnaround time of MTB culture would not be in favor of rapid MTB resistance analysis. Recently, several retrospective studies have demonstrated the efficacy of deep amplicon sequencing for culture-free MTB drug resistance detection using Deeplex Myc-TB platform with excellent performance among selected AFB-smear-positive clinical specimens (Kayomo et al., 2020; Bonnet et al., 2021; Jouet et al., 2021). However, with the low sensitivity of AFB smear test, a large proportion of MTB positive specimens with lower bacterial load could be missed, thus reducing the feasibility for rapid diagnosis of most drug-resistant specimens in actual clinical settings. A robust platform which is suitable for both AFB smear-positive and negative respiratory specimens is therefore in need to implementing the platform into routine clinical practice.

Our previous study introduced a highly sensitive and specific in-house IS6110-qPCR protocol for routine TB diagnosis with comparable performance to commercial platforms (Leung et al., 2018). In our practice, DNA extracts from IS6110-qPCR positive sputum were further tested for drug resistance-related mutation by Genotype MTBDR*plus* and MTBDR*sl* v2 (Hain Lifescience, Germany; MTBDR assay) for resistance-related mutation detection against RIF, INH, fluoroquinolones, and aminoglycosides. Sanger sequencing was performed on indeterminate results, such as specimens with absence of both wild-type probes and mutant probes (Lau et al., 2011; Siu et al., 2011a,b; Tam et al., 2019). Due to the high MDR-TB burden in our locality, false-resistant and false-susceptible cases could periodically be reported by line probe assay as a result of the vast variety of mutations in drug resistance-related genes (Javed et al., 2018). Although the discrepancies could be resolved by Sanger sequencing, the process is usually slow and labor intensive. To circumvent the problem, we recently developed a target amplicon sequencing protocol (TB-NGS) specifically targeting drug resistance-related genes in MTB isolates, which demonstrated complete concordance to conventional drug susceptibility testing (Tafess et al., 2020).

In order to reduce the turnaround time for drug-resistant MTB diagnosis, we extended TB-NGS into our rapid diagnostic platform to respiratory specimens. A total of 17 genes covering drug-resistant-related mutations of 8 classes of commonly used anti-TB drugs were selected, amplified and sequenced. As whole-genome data was not required by TB-NGS, data per sample could be greatly reduced and multiple specimens could be simultaneously analyzed in a single flow cell. The overall cost and turnaround time could be significantly reduced with the capability of direct application on respiratory specimen. In addition, we combined TB-NGS with our previously published IS6110-qPCR MTB diagnostic assay (Leung et al., 2018). The new routine diagnostic pipeline allows medium- to large-scale sample screening in daily clinical practice. For complicated TB cases such as retreatment cases and patients refractory to first-line treatment, TB-NGS could be performed to provide extra guidance.

In this study, we focused on the clinical utility of incorporating TB-NGS platform into routine settings. We performed a large-scale evaluation of TB-NGS workflow among respiratory specimens. Using respiratory specimens collected in Hong Kong and Guangzhou, the diagnostic performance of TB-NGS was assessed and compared with reference to the best available reference standard in our study cohort.

## Materials and methods

### Specimen collection and processing

From January 2018 to November 2019, respiratory specimens were collected from patients suffering from lower respiratory infections from 13 local chest clinics and 3 public hospitals in Hong Kong. Similarly, respiratory specimens were available from KingMed Diagnostic Centre for patients suspected of chest infections in Guangzhou, China. Each sputum specimen was decontaminated by N-Acetyl-L-Cysteine (NALC)-NaOH method with a final NaOH concentration of 2%. Approximately 500 μl processed sediments were inoculated onto Lowenstein-Jensen (LJ) slant (BioMérieux, Marcy l’Etoile, France) and BACTEC^™^ MGIT^™^ 960 Mycobacterial Detection System (Becton Dickinson, Baltimore, MD, United States), and specimen DNA were extracted from 500 μl processed sediments as previously described (Chan et al., 1996), and the identity of the positive AFB culture were confirmed by 16S rRNA sequencing (Yam et al., 2006).

### Crude DNA extraction, MTBC identification and line probe assay

For each specimen, crude DNA samples were extracted by AMPLICOR® Respiratory Specimen Preparation kit (Roche Lifescience, Germany). *Mycobacterium tuberculosis* complex (MTBC) detection was performed by IS6110-qPCR assay as previously described (Leung et al., 2018). Positive IS6110-qPCR was regarded when Ct value was less than 24.14. IS6110-qPCR positive specimens were further screened by MTBDR assay according to manufacturer’s instructions.

### Target amplification and preparation

Suspected genotypic resistant specimens were selected for TB-NGS. Specimens were purified by Agencourt AMPure XP PCR kit (Beckman Coulter, United States) in crude DNA extract to bead solution ratio of 1:1.8. Purified DNA was resuspended in 25 μl DNase-free water and stored at −80°C prior to usage. A total of 17 drug resistance-related gene targets (Table 1) were selected and amplified in five multiplex reaction mixes with primers listed in our previous study (Supplementary material; Tafess et al., 2020). A DNase-free water sample was added to each run as a negative control to minimize cross contamination.

**Table 1:** Gene targets for target amplicon sequencing.

**Drug**	**Gene**	**Amplicon size (bp)**	**Major mutation covered** ^*^
**Rifampicin**	*rpoB-*RRDR^1^	288	*rpoB* RRDR
	*rpoB*-full^1^	1,311	*rpoB* RRDR, V146F, I572F
**Isonizid**	*katG*	435	*katG* S315T
	*inhA* promoter	454	*inhA* C-15 T
	*inhA* structural	922	*inhA* codon 94 and 95
	*katG-furA* intergenic region	892	*furA* codon 4, −134 bp upstream deletion
**Ethambutol**	*embB*	955	*embB* M306V/I, G406A/D/S
	*ubiA*	1,119	Compensatory mutation
**Pyrazinamide**	*pncA*	813	Entire gene
	*rpsA*	1,601	Entire gene
**Fluoroquinolones**	*gyrA*	751	90–94 QRDR
	*gyrB*	1,054	N538D/E540V
**Aminoglycosides**	*eis* promoter	593	C-14 T,C-12 T, G-10A, G-10C
	*rpsL*	472	K43R/K88Q
	*rrs*	1,211	C1400,A1401/C1483
**Capreomycin**	*tylA*	945	R3^*^; Q22^*^ Loss of tlyA expression
**Linezolid**	*rplC*	710	G2061T/G2576T
	*rrl*	1,102	T460C

1 Two sets of primers were designed for rpoB to ensure a better coverage at 81 bp RIF resistance determining regions. *In accordance with 03–2018 literature review database.

Amplicons were purified again by AMPure XP PCR kit according to manufacturer’s instructions. DNA quantity was measured by Qubit dsDNA HS assay kit. Equal mass of DNA from each reaction mix was added to a new microcentrifuge tube.

### Library preparation and next-generation sequencing

Library was prepared using NEBNext^®^ Ultra^™^ II DNA Library Prep Kit for Illumina^®^ (New England Biolabs, United States) according to manufacturer’s instructions. Approximately 100 ng of the mixed purified amplicon was inputted, tagmented and barcoded. MiSeq was performed using MiSeq Reagent Kit v2 Nano kit, which generates 500Mbp output in 2 × 250 bp format. Each flow cell generates sequences for 24 samples.

### Bioinformatic analysis

To identify mutations from target amplicon sequencing data, fastq files were uploaded onto Bacteriochek (Advanced Biological Laboratories S.A., Luxembourg), a CE-IVD online platform for resistance detection. Minor variant mutations cutoffs were set at 20% and resistance-related mutation patterns were identified in accordance with 03–2018 literature review database. Successful sequencing results were defined according to the default criteria set by Bacteriochek followed by sufficient sequencing depth (≥500X) in *rpoB, katG, mabA, inhA, furA, gyrA, gyrB, rrs* and *eis* genes. These genes were selected as the surrogate markers for TB-NGS due to their importance in identifying MDR-TB and XDR-TB cases.

### Phenotypic DST

All MTBC culture-positive specimens were subjected to phenotypic drug susceptibility test (DST) using MGIT tubes with recommended critical concentration according to World Health Organization guidelines (World Health Organization, 2018). The critical concentrations for RIF, INH, ethambutol, streptomycin, pyrazinamide (PZA), kanamycin, amikacin, capreomycin, levofloxacin, moxifloxacin, linezolid are 1.0 μg/ml, 0.1 μg/ml, 5.0 μg/ml, 1.0 μg/ml, 100 μg/ml at pH 5.6, 2.5 μg/ml, 1.0 μg/ml, 2.5 μg/ml, 1.0 μg/ml, 0.25 μg/ml and 1.0 μg/ml, respectively. In brief, a loopful of mycobacterial colonies was suspended in 4 ml of BBL MiddleBrook 7H9 Broth (Becton Dickinson, Baltimore, MD, United States) containing 8–10 glass beads. The suspension was vortexed for 2–3 min to ensure a homogenized suspension. The suspension was then adjusted to a 0.5 McFarland turbidity standard. A total of 1 ml of the adjusted suspension was diluted into 4 ml sterile saline (referred as DST inoculum). Subsequently, a 1:10 growth control suspension for PZA and a 1:100 Growth control suspension for other antibiotics was prepared. Standard inoculum of both GC and DST inoculum was aseptically pipetted into a drug free MGIT tube and a drug containing MGIT tubes, respectively. The tubes were mixed thoroughly by gentle inversion for 3 to 4 times. They were then being inserted into a MGIT DST set carrier and placed into MGIT 960 instrument to incubate at 37°C for a maximum of 21 days with the exception of PZA being 14 days.

### Statistical analysis

IS6110-qPCR assay results were primarily compared with AFB culture results. Specimens with positive IS6110-qPCR assay results but negative AFB cultures were resolved by additional culture result from specimens collected on consecutive days. Indeterminate results were classified as cases with the absence of both IS6110 and IC amplification. These indeterminate samples were enumerated but were excluded from sensitivity and specificity calculations. Sensitivities, specificities, positive predictive values (PPV) and negative predictive values (NPV) were calculated with reference to the diagnostic results based on bacteriological and clinical information. TB-NGS results were compared primarily with reference to phenotypic DST. For minor variant cases, patients were closely monitored for another 6 months. Sequencing successful rate, result concordance and associated mutations were analyzed accordingly. Turnaround time of this study was calculated from sample collection date to report date.

## Results

### Specimen collection and preliminary screening result

From January 2018 to November 2019, 4,047 respiratory specimens from 2,627 patients were collected in Hong Kong and Guangzhou. Among these specimens, 473/4047 (11.7%) specimens showed positive MTBC culture, with 93/473 (19.7%) being AFB smear-positive. Mean turnaround time from sample collection to phenotypic DST was 50 days.

### IS6110-qPCR

Our previous study confirmed the cutoff Ct value for IS6110-qPCR assay was optimal at 24.14. Using Ct = 24.14 as cut off, 501/4047 (12.3%) specimens were identified as MTBC positive by IS6110-qPCR assay with Ct ranged from 4.17–24.13. The remaining 3454/4047(87.6%) were reported as MTB negative by IS6110-qPCR assay. A total of 92 specimens were reported as indeterminate result with both negative IS6110 and IC probe.

Using AFB culture as reference standard, IS6110-qPCR successfully detected 463/473 (97.9%) specimens with positive MTBC culture. For the remaining MTBC culture-positive specimens with negative IS6110-qPCR results, they showed positive IS6110 amplification with Ct ranged from 25.71 to 29.48. They were still reported as MTBC negative as the Ct value were greater than the cutoff Ct value. It is noted that 28 specimens showed positive IS6110-qPCR amplification but with negative AFB culture results. While these 28 specimens could be resolved with additional clinical information such as chest radiograph abnormalities compatible with pulmonary TB or other demographic features such as responses to anti-TB treatment and past TB history, they were still regarded as false positive cases due to negative AFB culture and the unavailability of culture isolated for further confirmation by 16S rRNA sequencing. Sensitivity and specificity of IS6110-qPCR assay were hence calculated at 100 and 100% for AFB specimens, and 97.4 and 99.2% for AFB smear negative specimens, respectively (Table 2).

**Table 2:** Diagnostic performance of IS6110-qPCR among 4,047 respiratory specimens using cutoff Ct value at 24.14.

		IS6110 qPCR			Resolved performance (%) [95% CI]			
AFB Smear	MTBC culture	Positive	Negative	Indeterminate	Sensitivity	Specificity	PPV	NPV
Positive (*n* = 97)	MTBC Positive (*n* = 93)^a^	93	0	0	100	100	100	100
	MTBC Negative (*n* = 4)	0	0	4				
Negative (*n* = 3,950)	MTBC Positive (*n* = 370)^a^	360	10	0	97.3 [95.1–98.7]	99.2 [98.8–99.5]	92.8 [89.9–94.9]	99.7 [99.5–99.8]
	MTBC Negative (*n* = 3,580)	28	3,464	88				
	Clinical TB cases (*n* = 28)^b^	28	0	0				
	Culture negative (*n* = 3,552)	0	3,376	88^c^				

a MTB culture-positive specimens from original or subsequent specimens collected in consecutive days.

b Clinical TB cases were defined as patients with abnormal chest radiographs compatible with pulmonary TB, or supported by other demographic features such as response to anti-TB treatment or patients with past TB history.

c Indeterminate cases were yielded due to the absence of both IS6110 and internal control amplification.

### MTBDR assay

All IS6110-qPCR positive specimens were tested by MTBDR assay with 438/501 (87.4%) specimens achieving interpretable results. The remaining 63/501 (12.6%) specimens showed uninterpretable result due to the absence of control TB probe. Among the positive specimens, 413/438 (94.5%) were reported with wild-type genotype, while mutant probe was detected in 25/438 (5.5%) specimens (Supplementary material).

### TB-NGS and concordance

TB-NGS was performed on all 25 mutant specimens detected by MTBDR assay. To investigate the efficacy of TB-NGS on wild-type genotypes, 67 wild-type specimens were randomly selected with IS6110-qPCR Ct ranged from 6.35–24.1, yielding a total of 92 specimens enrolled for TB-NGS analysis. Using the criteria, we defined, 83/92 (90.2%) specimens were successfully sequenced. The average sequencing depth for each specimen is 22,500X (95% confidence interval: 20660X, 24,400X). Resistant-related mutations were found in 30 specimens with mutation patterns listed in Table 3 and Supplementary material.

**Table 3:** Summary of drug-resistant mutations detected by TB-NGS.

Drugs	Gene	Mutation patterns	No. of specimens	Mutation frequency (±SD)
Rifampicin	*rpoB*	D516V	1	99.22
		D516Y^a^	1	31.55
		S531L	7	84.97 (±17.28)
		H526N^c^	2	95.91 (±1.50)
		H526L	1	100
		H526R	1	66.96
		H526R^b^	1	20.94
		I572L^c^	2	98.28 (±1.69)
		I572F	1	98.5
Isoniazid	*katG*	S315T	16	98.67 (±1.96)
		S315N	2	98.70 (±0.01)
	*mabA*	c-15t^d^	2	99.70 (±0,43)
	*inhA*	I194T^d^	1	98.5
	*furA-inhA*	None	N/A	N/A
Ethambutol	*embB*	M306V	3	99.69 (±0.26)
		M306I	3	89.05 (±15.35)
		G406S	3	99.45 (±0.95)
		G406C	1	98.87
	*ubiA*	None	N/A	N/A
Pyrazinamide	*pncA*	V139G	2	98.665 (±0.66)
	*rpsA*	None	N/A	N/A
Fluoroquinolones	*gyrA*	A74S^e^	1	35.90
		A90V	2	98.55 (±0.32)
		D94G	3	80.10 (±16.66)
		D94N	2	100.0 (±0.01)
	*gyrB*	None	N/A	N/A
Streptomycin	*rpsL*	K43R	6	98.36
		K88R	2	98.87 (±0.04)
	*rrs*	c513t	1	99.63
Aminoglycosides	*rrs*	a1401g	1	100.0
	*eis*	None	N/A	N/A
Capreomycin	*tlyA*	None	N/A	N/A
Linezolid	*rrl*	None	N/A	N/A
	*rplC*	None	N/A	N/A

Abbreviation: SD: standard deviation; N/A, not available.

a One strain (WC30) carried a minor variant rpoB D516Y with low mutation frequency at 31.55% as detected by TB-NGS.

b One strain (WC29) carried a minor variant rpoB H526R with low mutation frequency at 20.94% as detected by TB-NGS.

c Two strains carried both rpoB H526N and I572L mutation. Phenotypic DST result later showed that the two strains were phenotypically resistant to RIF.

d One strain carried both mabA c-15 t and inhA I194T mutation. Phenotypic DST result later revealed that the strain was phenotypically resistant to INH.

e One strain (WC28) carried a minor variant gyrA A74S with low mutation frequency at 35.9% as detected by TB-NGS.

TB-NGS demonstrated 100% concordance among all 25 drug-resistant strains. Among these strains, one INH mono-resistant specimen (WC14) defined by MTBDR assay was confirmed as MDR-TB by TB-NGS with *rpoB* I572F mutation located outside RIF resistance determining region (RRDR). The results were further confirmed by phenotypic DST as MDR-TB. One genotypic FQs mono-resistant specimen (WC10) was also identified with *gyrA* D94G missense mutation and had concordant result between MTBDR assay, TB-NGS and phenotypic DST. Meanwhile, 5 additional wild-type strains defined by MTBDR assay were found with drug resistance-related mutations in TB-NGS. Among these five specimens, two (WC26 and WC27) were genotypically STR mono-resistant with *rpsL* K43R. The remaining three specimens were defined as minor variants by TB-NGS but were considered as wild-type strains by MTBDR assay. Two *rpoB* mutations D516V (WC30) and H526R (WC29) were separately identified in two specimens with a low mutation frequency of 31.55 and 20.94%, respectively (Table 3). One specimen (WC28) was found to carry *gyrA* mutation A74S with mutation frequency of 35.90%. Consistent results were obtained in technical replicates, which confirmed the presence of the mutation despite at a low frequency.

### Phenotypic DST

A total of 68/92 specimens enrolled in TB-NGS study yielded MTBC culture-positive specimens and were subjected to phenotypic DST (Supplementary material). Among these 68 specimens, 6 phenotypically pan-susceptible strains yielded indeterminate TB-NGS results and were excluded from subsequent analysis. In the remaining 62 strains, 12 were drug-resistant TB with resistance to one or more anti-TB drugs, 4 were MDR-TB, 6 were pre-XDR-TB, and 40 were pan-susceptible strains (Table 4). All remaining 408 MTBC culture-positive specimens were defined as pan-susceptible by phenotypic DST.

**Table 4:** Phenotypic DST profiles of MTBC culture-positive strains from Hong Kong and Guangzhou by phenotypic DST in this study.

**Specimen type**	**Resistance pattern defined by TB-NGS**	**No. of specimens**	**Phenotypic drug susceptibility patterns**									
			**culture (+)**	**RIF**	**INH**	**EMB**	**PZA**	**STR**	**FLQ**	**CAP**	**AMI**	**LZD**
Genotypic drug resistance defined by MTBDR assay (*n* = 25)	Drug-resistant TB (*n* = 15)	2	1	R	S	S	S	S	S	S	S	S
		1	1	R	S	S	S	R	S	S	S	S
		5	2	S	R	S	S	S	S	S	S	S
		1	1	S	R	R	S	S	S	S	S	S
		1	1	S	R	R	S	R	S	S	R	S
		4	3	S	R	S	S	R	S	S	S	S
		1	1	S	S	S	S	S	R	S	S	S
	MDR-TB (*n* = 4)	2	2	R	R	R	R	S	S	S	S	S
		2	2	R	R	S	S	R	S	S	S	S
	Pre-XDR-TB (*n* = 6)	2	2	R	R	R	S	S	R	S	S	S
		4	4	R	R	R	S	R	R	S	S	S
Drug resistance not detected by MTBDR assay (*n* = 476)	Drug-resistant TB (*n* = 2)	2	2	S	S	S	S	R	S	S	S	S
	Minor variant (*n* = 3)^a^^,^ ^b^^,^ ^c^	3	3	S	S	S	S	S	S	S	S	S
	Pan-susceptible TB (*n* = 53)	53	37	S	S	S	S	S	S	S	S	S
	Unsuccessful sequencing (*n* = 9)	9	6	S	S	S	S	S	S	S	S	S
	TB-NGS not performed (*n* = 409)	409	405	S	S	S	S	S	S	S	S	S

Abbreviation: RIF, rifampicin, INH, isoniazid; EMB, ethambutol, PZA, pyrazinamide; FLQ, fluoroquinolones; STR, streptomycin; AMIs, aminoglycosides; CAP, capreomycin; LZD, linezolid

a WC29 carried a minor variant at rpoB H526R with mutation frequency of 20.94% by TB-NGS. Phenotypic DST result showed that the strain was susceptible to RIF.

b WC30 carried a minor variant at rpoB D516Y with mutation frequency of 31.55% by TB-NGS. Phenotypic DST result showed that the strain was susceptible to RIF.

c WC28 carried a minor variant at gyrA A74S with mutation frequency of 35.90% by TB-NGS. Phenotypic DST result showed that the strain was susceptible to FQs.

Using phenotypic DST as reference standard, TB-NGS showed concordant results in 59/62 specimens. The three discordant cases were pan-susceptible by phenotypic DST (Table 4, Footnote a–c) and was identified by TB-NGS as minor variant cases with a mutation frequency less than 35%. These patients exhibited no signs of drug resistance development during 6 month follow-up sessions, and were subsequently discharged after directly observed short course treatment (DOTS).

## Discussion

Conventional TB diagnosis is confirmed fundamentally on AFB culture and subsequent phenotypic DST. However, the entire procedures from sample collection to phenotypic DST require at least 5–12 weeks. Due to the long turnaround time of MTB culture, results are usually unavailable to clinicians for treatment guidance when starting empirical treatments. The long turnaround time is more evident among drug-resistant specimens due to the even slower growth of resistant MTB strains. Molecular approaches such as line-probe assays are timely and reliable genotypic DST methods, but increasing number of studies showed that false resistance could sometimes be reported due to the presence of naturally occurring polymorphisms (Coll et al., 2015; Hu et al., 2019). In addition, the presence of uncommon mutation might also result in discordant genotypic and phenotypic drug susceptibility results. For instance, MTB strains with *rpoB* L533P mutation might exhibit slightly elevated RIF Minimum Inhibitory concentration compared to fully susceptible strains but remain phenotypically susceptible when RIF critical concentration at 1 μg/ml was performed in MGIT SIRE as recommended by WHO guidelines (Tam et al., 2017; Shea et al., 2021). Similarly, while mutation at promoter region of *inhA* is generally believed to cause low-level INH resistance, concomitant mutation at *inhA* I194T could drastically increase INH resistance, rendering the drug useless even when a higher INH dosage was used (Machado et al., 2013). A rapid and reliable diagnostic method that reveals the identity of drug resistance-related mutation is thus urgently needed to fully explore the identity of the mutation.

The basis of TB-NGS was developed according to our previous study, which has demonstrated excellent reliability in identifying drug resistance-related mutations among culture isolates (Tafess et al., 2020). To extend the test to respiratory specimens, several modifications were implemented. First of all, instead of multiplex PCR of 17 gene regions in a single-tube, target amplification was separated into five multiplex PCR with each tube amplifying 4–5 regions of similar sizes to improve the amplification efficiency. To improve the sensitivity of TB-NGS especially on RIF, an additional target at *rpoB* covering RRDR and codon I572 was included. With these modifications, TB-NGS successfully sequenced 83/92 (90.2%) of the specimens enrolled in this study with 100% concordance when compared with phenotypic DST. For the remaining 9 specimens with unsuccessful TB-NGS results, they were all AFB smear-negative specimens with a Ct value ranged from 19.57–24.1. Meanwhile, most of the sequenced specimens have IS6110 Ct value of less than 18, indicating that the detection limit of TB-NGS would be equivalent to approximately 250 CFU/ mL according to the LOD analysis performed in our previous study (Leung et al., 2018). The higher demand in bacterial load might also explain the nine specimens with positive IS6110-qPCR but indeterminate TB-NGS results in this study.

Based on the TB-NGS result several uncommon mutation patterns were identified. One specimen (WC14) exhibited concurrent INH-resistance-related mutations at both *inhA* regulatory region c-15 t and structural region I194T with mutation frequency of 99.39 and 98.50%, respectively. Phenotypic DST revealed that the specimen was resistant to INH at 0.4 μg/ml, indicating high-level INH resistance instead of the low-level resistance commonly reflected by single mutation at *inhA* regulatory site. In addition, two MDR-TB specimens from the same patient (WC11and WC12) exhibited concurrent *rpoB* H526N and I572L mutations with mutation frequency of >95%. Mutation at *rpoB* H526N is normally regarded as a natural polymorphism (Jamieson et al., 2014), while *rpoB* I572L is a RIF resistant-related mutation located outside RRDR and is commonly not recognized by GeneXpert MTB/RIF Ultra and MTBDR*plus* assay. Similarly, one INH mono-resistant specimen defined by MTBDR assay was confirmed as MDR-TB by TB-NGS with *rpoB* I572F mutation. For PZA resistance detection, two PZA resistant specimen from one patient were identified in our study cohort. TB-NGS showed that the strain carried *pncA* V139G mutation which is a PZA resistance-related mutation commonly found in our locality as previously reported (Tam et al., 2019). The resistance profiles identified by TB-NGS in this study highlighted the strength of our diagnostic platform. Being an unbiased platform for identifying drug resistance mutations, TB-NGS could provide a much more comprehensive analysis on the resistance pattern of each specimen with a much shorter turnaround time. This would facilitate a judicious use of antibiotics especially in the treatment of MDR-TB.

In this study, three quasispecies were identified among wildtype specimens, of which two were *rpoB* mutations that could be correlated to phenotypic RIF resistance. These minor variants were consistent in multiple technical replicates, suggesting that these minor variants were unlikely to be sequencing artifacts. Phenotypic DST on these three quasispecies revealed pan-susceptible phenotypes, and the patients fully recovered after 6-months DOTS follow-up without complications. With a mutation frequency of less than 35%, these minor variants could be well distinguished from dominant resistant mutations which usually carried a mutation frequency of >60%. Therefore, these minor variants would not complicate empirical clinical judgment. Nevertheless, to fully elucidate the effect of these minor variants, a large-scale longitudinal study could be required to determine the progression of minor variant resistance developments.

**Figure 1:**
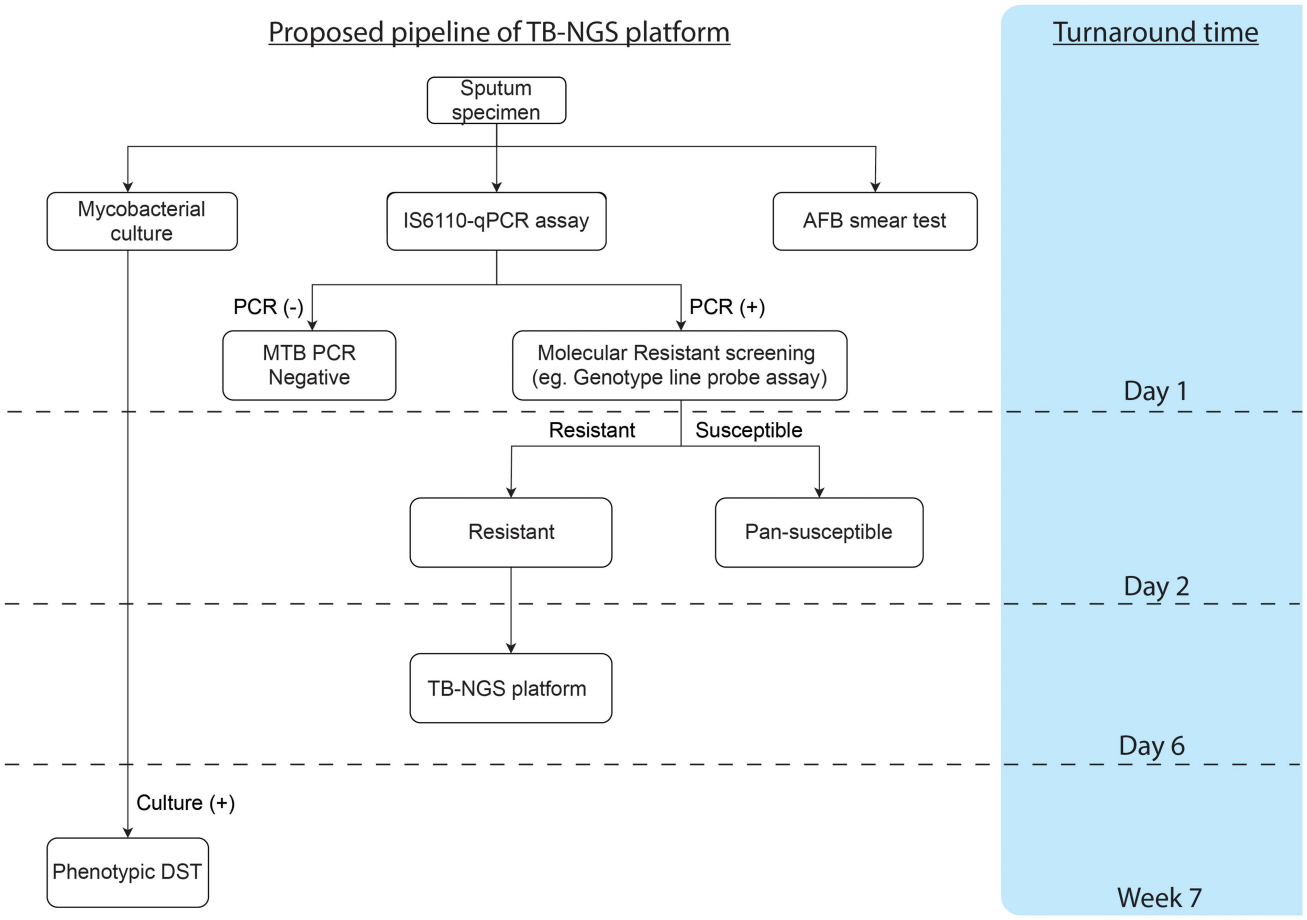
Proposed workflow and cumulative turnaround time for each procedure estimated from specimen collection.

TB-NGS generally requires a minimum turnaround time of 6 working days. Depending on the Illumina flow cell being used, TB-NGS can accommodate 12–96 samples per single run, which would fit low to high throughput analysis. Furthermore, with Bacteriochek having CE-IVD systems, the assay would be well suited in most clinical laboratories. While the platform is suitable for routine surveillance in low endemic regions in developed countries, a cost-effective protocol is highly recommended for high endemic regions as demonstrated in our study (Figure 1). Preliminary screening can first be performed for MTBC detection, followed by molecular genotypic resistance detection. For cases with resistant mutation or upon clinical suspicions, such as patients unresponsive to treatment or requiring additional support among treatment defaulters and retreatment cases, TB-NGS can be performed to provide a comprehensive drug resistance profile. Applying this algorithm to our study, only 25 out of the 4,000 specimens would require additional aid from TB-NGS. More importantly, our diagnostic algorithm missed no drug-resistant case as indicated by phenotypic DST results on all MTBC culture-positive specimens. The implementation of our proposed algorithm would therefore greatly reduce the workload and financial burden in clinical microbiology laboratories, especially in high endemic regions with limited resources.

Despite the potential of TB-NGS for better MTB management support, the assay has its limitations. The current platform is based on the knowledge of resistance-related mutation. For novel mutations located outside targeted sites they will not be detected. Nevertheless, the current test has included all well-established resistance-conferring mutations which would have been suitable for normal routine clinical practice. On the other hand, while the guideline updated in late 2020 included bedaquiline as one of the medications for XDR-TB definition, our current TB-NGS protocol did not include bedaquiline genotypic resistance test due to several reasons. As a new anti-TB drug recommended by WHO in 2016, the exact mechanism for bedaquiline resistance pattern in clinical isolates are still far from fully elucidated (Nguyen et al., 2018; Nieto Ramirez et al., 2020; Gomez-Gonzalez et al., 2021). In addition, the occurrence of bedaquiline resistance TB strains in our locality is still extremely rare. Among 4,043 specimens collected in this study, only 6 pre-XDR-TB specimens were identified while still susceptible to PZA, LZD and aminoglycosides. Genotypic resistance test would still be necessary in further development of TB-NGS platform due to increasing use of bedaquiline in complicated TB cases as well as the emergence of bedaquiline resistant strains (Chesov et al., 2021). Lastly, while other sequencing platforms such as Nanopore might be a more rapid and budget-friendly approach, we chose Illumina as the primary sequencing platform due to its high single read accuracy for the investigation of minor variants in our study cohort. Evaluation of TB-NGS by Nanopore in a multi-center analysis is therefore warranted to further extend the utility of TB-NGS for more flexible and cost-effective routine service.

To conclude, TB-NGS is an accurate platform for TB drug resistance detection. When accompany with our proposed diagnostic algorithm, the workflow would provide a cost-effective diagnostic solution for complicated TB cases and allow early detection of specimens with potential drug resistance developments.

## Data availability statement

The data presented in this study are deposit in NCBI database, Accession Number SAMN30429057-SAMN30429139.

## Ethics statement

This study has been approved by the Institutional Review Board of the University of Hong Kong/Hospital Authority Hong Kong West Cluster (Ref. number: UW 12-309).

## Author contributions

KL, KT, TN, and GS were responsible for designing the study, performing the experiments and writing the manuscript. KL was responsible for bioinformatic and statistical analysis of this study. H-YL and RS were responsible for doing the experiments. OM, S-HY, J-XC, and QH were responsible for providing processing specimens from mainland China. W-CY is the corresponding author and was responsible of supervising the study. All authors contributed to the article and approved the submitted version.

## Funding

This study is supported by the Science and Technology Planning Project of Guangdong Province, China (grant number: 2019B121205010).

## Conflict of interest

OM, S-HY, J-XC and QH were employed by KingMed Diagnostics.

The remaining authors declare that the research was conducted in the absence of any commercial or financial relationships that could be construed as a potential conflict of interest.

## Publisher’s note

All claims expressed in this article are solely those of the authors and do not necessarily represent those of their affiliated organizations, or those of the publisher, the editors and the reviewers. Any product that may be evaluated in this article, or claim that may be made by its manufacturer, is not guaranteed or endorsed by the publisher.

## Abbreviations

TB: Tuberculosis;
MTB,: *Mycobacterium tuberculosis;*
MDR-TB,: Multidrug-resistant TB;
RIF,: Rifampicin;
INH,: Isoniazid;
RR-TB,: RIF mono-resistant;
TB-NGS,: target amplicon sequencing protocol;
NALC,: N-Acetyl-L-Cysteine;
LJ,: Lowenstein-Jensen;
MTBC,: *Mycobacterium tuberculosis* complex;
RRDR,: RIF resistance determining region;
DOTS,: Directly observed short course treatment;
NGS,: Next-generation sequencing
