# Supplementary material for: Clinical utility of target amplicon sequencing test for rapid diagnosis of drug-resistant Mycobacterium tuberculosis from respiratory specimens
Source: Front Microbiol. 2022 Sep 9;13:974428. doi: 10.3389/fmicb.2022.974428 (PMC9505518; doi:10.3389/fmicb.2022.974428)
Supplement: Supplementary file 1 [file Data_Sheet_1.docx]

**Clinical utility of target amplicon sequencing test for rapid diagnosis of drug resistant *Mycobacterium tuberculosis* from respiratory specimens**

*Kenneth Siu-Sing Leung^a1^, Kingsley King-Gee Tam^a1^*, *Timothy Ting-Leung Ng^a2^*, Hiu-Yin Lao^2^, Raymond Chiu-Man Shek^1^, Oliver Chiu Kit Ma^3^, Shi-hui Yu^5^, Jing-xian Chen^4^, Qi Han^4^, Gilman Kit-Hang Siu^2^, Wing-Cheong Yam^1^*

^a.^ These authors contribute equally to this study.

**Author Affiliations:**

^1^Department of Microbiology, Queen Mary Hospital, The University of Hong Kong, Hong Kong Special Administrative Region, China

^2^Department of Health Technology and Informatics, The Hong Kong Polytechnic University, Hong Kong Special Administrative Region, China

^3^ KingMed Diagnostics, Science Park, Hong Kong Special Administrative Region, China

^4^ Guangzhou KingMed Diagnostics Group, Guangzhou, China

^5^ Guangdong-Hong Kong-Macao Joint Laboratory of Respiratory Infectious Disease, China

^*^Corresponding author. Department of Microbiology, Queen Mary Hospital, The University of Hong Kong, Hong Kong Special Administrative Region, China. Tel: +852-22554821; Fax: +852-28551241; E-mail: [wcyam@hkucc.hku.hk](mailto:wcyam@hkucc.hku.hk)

**Abbreviations**

Tuberculosis (TB); *Mycobacterium tuberculosis* (MTB); Multidrug-resistant TB (MDR-TB); Rifampicin (RIF); Isoniazid (INH); RIF mono-resistant (RR-TB); target amplicon sequencing protocol (TB-NGS); N-Acetyl-L-Cysteine(NALC); Lowenstein-Jensen (LJ); *Mycobacterium tuberculosis* complex (MTBC); RIF resistance determining region (RRDR); Directly observed short course treatment (DOTS); Next generation sequencing (NGS)

**Supplementary material**

Each multiplex reaction is a 25µL mix consisting of 1X Q5 buffer (New England Biolab, USA), 1X GC enhancer (New England Biolab, USA), 200µM dNTP, 0.25µL Q5 polymerase (New England Biolab, USA), 2.5µL of the corresponding primer mix and 2µL purified crude DNA extract. PCR condition was set as follow: initial denaturation at 98°C for 10 seconds, followed by 10 cycles of touch-down amplification stage: 98°C for 10 seconds, annealing for 30 seconds from 65.5°C to 61°C with each cycle dropped by 0.5°C, and elongation at 72°C for 45 seconds. The third amplification stage was set with 98°C for 10 seconds, annealing at 60.5°C for 30 seconds and elongation at 72°C for 45 seconds. Finally, final extension was set at 72°C for 7 minutes.

**Data information**

The data presented in this study are deposit in NCBI database, Accession Number SAMN30429057- SAMN30429139.

**Supplementary Table S1A** Primer mix of reaction 1

| Target | Volume (µL) | |
| --- | --- | --- |
|  | 20µM Forward primer | 20µM Reverse primer |
| *rpoB-*RRDR | 10 | 10 |
| *katG* | 10 | 10 |
| *rpsL* | 10 | 10 |
| *eis* | 10 | 10 |
| *rplC* | 10 | 10 |
| **Total volume** | **100** **µL** | |

**Supplementary Table S1B.** Primer mix of reaction 2

| Target | Volume (µL) | |
| --- | --- | --- |
|  | 10µM Forward primer | 10µM Reverse primer |
| *mabA* | 12.5 | 12.5 |
| *gyrA* | 12.5 | 12.5 |
| *pncA* | 12.5 | 12.5 |
| *inhA-structural* | 12.5 | 12.5 |
| **Total volume** | **100 µL** | |

**Supplementary Table S1C.** Primer mix of reaction 3

| Target | Volume (µL) | |
| --- | --- | --- |
|  | 20µM Forward primer | 20µM Reverse primer |
| *furA* | 10 | 10 |
| *tlyA* | 10 | 10 |
| *gyrB* | 10 | 10 |
| *rrs* | 10 | 10 |
| *rpsA* | 10 | 10 |
| **Total volume** | **100 µL** | |

**Supplementary Table S1D.** Primer mix of reaction 4

| Target | Volume (µL) | |
| --- | --- | --- |
|  | 10µM Forward primer | 10µM Reverse primer |
| *embB* | 25 | 25 |
| *rpoB-full* | 25 | 25 |
| **Total volume** | **100 µL** | |

**Supplementary Table S1E.** Primer mix of reaction 5

| Target | Volume (µL) | |
| --- | --- | --- |
|  | 10µM Forward primer | 10µM Reverse primer |
| *rrl* | 25 | 25 |
| *ubiA* | 25 | 25 |
| **Total volume** | **100 µL** | |

| **Category** | **Specimen** | **AFB culture** | **Phenotypic DST profile^1^** | **MTBDR assay results** | **TB-NGS results** | | | | | | | | | | | | | | | | |
| --- | --- | --- | --- | --- | --- | --- | --- | --- | --- | --- | --- | --- | --- | --- | --- | --- | --- | --- | --- | --- | --- |
|  |  |  |  |  | ***rpoB*** | ***katG*** | ***inhA*** | ***mabA*** | ***furA*** | ***embB*** | ***ubiA*** | ***pncA*** | ***rpsA*** | ***gyrA*** | ***gyrB*** | ***rpsL*** | ***rrs*** | ***eis*** | ***tlyA*** | ***rrl*** | ***rplC*** |
| **Resistant [n=27]** | WC01 | + | RIF-R | *rpoB* WT7 (-) | H526L  (100) | None | I21V (100) | None | None | None | None | None | None | None | None | None | None | None | None | None | None |
|  | WC02 | - | N/A | *rpoB* WT7 (-) | H526R  (66.96) | None | None | None | None | None | None | None | None | None | None | None | None | None | None | None | None |
|  | WC03 | + | RIF-R, STR-R | *rpoB* WT8 (-); Mut3 (+) | S531L  (80.43) | None | None | None | None | None | V188A (9.71) | None | None | None | None | K43R (97.64) | None | None | None | None | None |
|  | WC04 | + | INH-R, STR-R | *katG* WT (-); Mut1 (+) | None | S315T (99.21) | None | None | None | None | None | None | None | None | None | K43R (97.38) | None | None | None | None | None |
|  | WC05 | + | INH-R, STR-R | *katG* WT (-); Mut1(+) | None | S315T (100) | None | None | None | None | None | None | None | None | None | K43R (99.7) | None | None | None | None | None |
|  | WC06 | + | INH-R, STR-R | *katG* WT (-); Mut1(+) | None | S315T (100) | None | None | None | None | None | None | None | None | None | K43R (99.09) | None | None | None | None | None |
|  | WC07 | - | N/A | *katG* WT (-); Mut1(+) | None | S315T (98.96) | None | None | None | None | None | None | None | None | None | C513T (99.63) | None | None | None | None | None |
|  | WC08 | + | INH-R, EMB-R, STR-R, AMI-R | *inhA* WT1 (-); Mut1(+),  *rrs* WT1 (-); Mut1(+) | None | None | None | c-15t (100) | None | M306V (100) | None | None | None | None | None | None | a1401g (100) | None | None | None | None |
|  | WC09 | + | INH-R, EMB-R | *katG* WT (-); Mut1(+) | None | S315T (92.58) | None | None | None | M306I (71.38) | V188A (5.37) | None | None | None | None | None | None | None | None | None | None |
|  | WC10 | + | LVX-R, OFX-R | *gyrA* WT3 (-); Mut3C(+) | None | None | None | None | None | None | None | None | None | D94G (93.51) | None | None | None | None | None | None | None |
|  | WC11 | + | RIF-R, INH-R, EMB-R, PZA-R | *rpoB* WT 7 (-),  *katG* WT (-); Mut1(+) | H526N (96.97)  I572L (99.47) | S315T (100) | None | None | None | M306I (98.13) | None | V139G(99.13) | None | None | None | None | None | None | None | None | None |
|  | WC12 | + | RIF-R, INH-R, EMB-R, PZA-R | *rpoB* WT 7 (-),  *katG* WT (-); Mut1(+) | H526N (94.85) I572L (97.08) | S315T (100) | None | None | None | M306I (97.70) | None | V139G(98.20) | None | None | None | None | None | None | None | None | None |
|  | WC13 | + | RIF-R, INH-R, STR-R | *rpoB* WT 8 (-); Mut3(+),  *katG* WT (-); Mut1(+) | S531L  (59.44) | S315T (99.18) | None | None | None | None | None | None | None | None | None | K43R (98.68) | None | None | None | None | None |
|  | WC14 | + | RIF-R, INH-R, STR-R | *inhA* WT1 (-); Mut1(+) | I572F  (98.5) | None | I194T (98.5) | C-15T (99.39) | None | None | None | None | None | None | None | K88R (98.9) | None | None | None | None | None |
|  | WC15 | + | RIF-R, INH-R, EMB-R, LVX-R, OFX-R | *rpoB* WT 8 (-); Mut3(+),  *katG* WT (-); Mut1(+)  *gyrA* WT3 (-); Mut3B(+) | S531L  (100) | S315T (100) | None | None | None | G406S (100) | None | None | None | D94N (100) | None | None | None | None | None | None | None |
|  | WC16 | + | RIF-R, INH-R, EMB-R, LVX-R, OFX-R | *rpoB* WT 8 (-); Mut3(+),  *katG* WT (-); Mut1(+)  *gyrA* WT3 (-); Mut3B(+) | S531L  (100) | S315T (100) | None | None | None | G406S (100) | None | None | None | D94N (100) | None | None | None | None | None | None | None |
|  | WC17 | + | RIF-R, INH-R, EMB-R, LVX-R, OFX-R, STR-R | *rpoB* WT 7 (-),  *katG* WT (-); Mut1(+)  *gyrA* WT3 (-); Mut3C(+) | S531L  (95.82) | S315T (99.36) | None | None | None | G406C (98.87) | None | None | None | D94G (87.4) | None | K43R (98.14) | None | None | None | None | None |
|  | WC18 | + | RIF-R, INH-R, EMB-R, LVX-R, OFX-R, STR-R | *rpoB* WT 3&4 (-); Mut1(+),  *katG* WT (-); Mut1(+),  *gyrA* WT2 (-); Mut1(+) | D516V  (99.22) | S315T (99.33) | None | None | None | M306V (99.56) | None | None | None | A90V (98.33) | None | K88R (98.84) | None | None | None | None | None |
|  | WC19 | + | RIF-R, INH-R, EMB-R, LVX-R, OFX-R | *rpoB* WT 3&4(-); Mut1(+),  *katG* WT (-); Mut1(+),  *gyrA* WT2 (-); Mut1(+) | S531L  (97.73) | S315T (99.24) | None | None | None | G406S (98.36) | None | None | None | A90V (98.78) | None | None | None | None | None | None | None |
|  | WC20 | + | RIF-R, INH-R, EMB-R, LVX-R, OFX-R | *rpoB* WT 7 (-)  *katG* WT (-); Mut1(+)  *gyrA* WT3 (-); Mut3C(+) | S531L  (63.68) | S315T (98.71) | None | None | None | M306V (99.52) | None | None | None | D94G (62.08) | None | None | None | None | None | None | None |
|  | WC21 | + | INH-R | *katG* WT (-); Mut1(+) | None | S315T (99.19) | None | None | None | None | None | None | None | None | None | K43R (99.25) | None | None | None | None | None |
|  | WC22 | - | N/A | *katG* WT (-); Mut1(+) | None | S315T (99.08) | None | None | None | None | None | None | None | None | None | None | None | None | None | None | None |
|  | WC23 | - | N/A | *katG* WT (-); Mut1(+) | None | S315T (95.32) | None | None | None | None | None | None | None | None | None | None | None | None | None | None | None |
|  | WC24 | + | INH-R | *katG* WT (-); | None | S315N (98.71) | None | None | None | None | None | None | None | None | None | None | None | None | None | None | None |
|  | WC25 | - | N/A | *katG* WT (-); | None | S315N (98.69) | None | None | None | None | None | None | None | None | None | None | None | None | None | None | None |
|  | WC26 | + | STR-R | WT | None | None | None | None | None | None | None | None | None | None | None | K43R (99.25) | None | None | None | None | None |
|  | WC27 | + | STR-R | WT | None | None | None | None | None | None | None | None | None | None | None | K43R (97.63) | None | None | None | None | None |
| **Minor variants [n=3]** | WC28 | + | S | WT | None | None | None | None | None | None | None | None | None | A74S (35.9) | None | None | None | None | None | None | None |
|  | WC29 | + | S | WT | H526R  (20.94) | None | None | None | None | None | None | None | None | None | None | None | None | None | None | None | None |
|  | WC30 | + | S | WT | D516Y  (31.6) | None | None | None | None | None | None | None | None | None | None | None | None | None | None | None | None |
| **Susceptible [n=37]** | N/A | + | S | WT | None | None | None | None | None | None | None | None | None | None | None | None | None | None | None | None | None |
| **Susceptible [n=16]** | N/A | - | N/A | WT | None | None | None | None | None | None | None | None | None | None | None | None | None | None | None | None | None |

**Table S2. Target amplicon sequencing results on 83 IS6110-qPCR (+) specimens**

**^1^** Phenotypic DST was performed using MGIT tubes with recommended critical concentration according to World Health Organization guidelines.

**Abbreviations:** Abbreviation: RIF, rifampicin, INH, isoniazid; EMB, ethambutol, PZA, pyrazinamide; FLQ, fluoroquinolones; LVX: levofloxacin, OFX: ofloxacin, STR, streptomycin;

AMIs, aminoglycosides; CAP, capreomycin; LZD, linezolid
